# Dataset of air velocity and temperature fields inside an insulated box equipped with phase change material under several operating conditions

**DOI:** 10.1016/j.dib.2023.109934

**Published:** 2023-12-09

**Authors:** Tanathep Leungtongkum, Denis Flick, Nattawut Chaomuang, Alain Denis, Onrawee Laguerre

**Affiliations:** a Université Paris-Saclay, INRAE, FRISE, 92761, Antony, France; b Université Paris-Saclay, INRAE, AgroParisTech, UMR SayFood, 91120 Palaiseau, France; c Department of Food Technology, Faculty of Science, Chulalongkorn University, Wangmai, Pathumwan, Bangkok, 10330, Thailand; d Department of Food Engineering, School of Engineering, King Mongkut's Institute of Technology Ladkrabang, Bangkok 10520, Thailand

**Keywords:** Heat transfer, Airflow, Insulated box, Phase change material, Food cold chain

## Abstract

This article contains a description of protocol to measure air velocity field (by Particle Image Velocimetry - PIV) and temperature field (by T-type thermocouples) in an insulated box equipped with Phase Change Material (PCM) of melting point 0 °C. The influence of various conditions was studied: i) PCM position (at sidewall and at top), ii) aspect ratio of the box (height/width ∼ 1 and 1.7), iii) ambient temperature (10 °C, 20 °C and 30 °C), iv) test product initial temperature (4 °C and 10 °C) and vi) spacing beneath the load (0 mm and 20 mm). This article is related to a published research paper, it provides the dataset of all experiments which can be useful for experimenter to understand the phenomena and for expert in numerical model to validate the developed model e.g., by Computational Fluid Dynamic.

Specifications Table

**Table:** 

Subject	Bioengineering
Specific subject area	Air velocity and temperature fields measured in an empty and loaded insulated box with cold source generated by Phase Change Material
Type of data	Table Image Graph MATLAB code Dataset
How the data were acquired	Air velocity measured by Particle Image Velocimetry (PIV) Temperature measured by calibrated thermocouples T-Type.
Data format	Raw Analysed
Description of data collection	Air velocity (at middle plane and lateral plane) and temperature (at middle plane) in insulated boxes equipped with PCM (melting point 0 °C) under various conditions: • PCM position (at sidewall and at top), • aspect ratio of box (height/width = 1 and 1.7), • ambient temperature (10 °C, 20 °C and 30 °C), • test product (Tylose, TYL) initial temperature (4 °C and 10 °C) • space beneath the load (0 mm and 20 mm).
Data source location	• INRAE (FRISE Research unit) • Antony • France
Data accessibility	With the article Repository name: Mendeley Data Data identification number: 10.17632/ggmkzk634h.1 10.17632/sz5dgkz7k8.1 Direct URL to data: https://data.mendeley.com/datasets/ggmkzk634h/1 https://data.mendeley.com/datasets/sz5dgkz7k8/1 Repository name: GitHub Data identification number: 10.5281/zenodo.6900688 Direct URL to data: https://github.com/Tanathepl/Temperature-contour.git
Related research article	Leungtongkum, T., Flick, D., Chaomuang, N., Denis, A., & Laguerre, O. (2023). Influence of use conditions on heat transfer in an insulated box equipped with a phase change material. Journal of Food Engineering, 357, 111644. https://doi.org/10.1016/j.jfoodeng.2023.111644

## 1 Value of the Data

• The presented data (air velocity, temperature and product temperature) allow the understanding of the physical phenomena (heat transfer and airflow) in a closed cavity with a cold source.
• These data are rare in literature because of the difficulty in measuring low air velocity in food transport in an insulated box. It requires an appropriate experimental setup.
• Data would be useful for understanding phenomena, insulated box design and optimal operating conditions to maintain product temperature at a recommended value along the supply chain.
• Data can be used to compare with the results of numerical models.

## 2 Data Description

Data presented in this article include raw data of air velocity and temperature measurement, figures of air velocity field and temperature contour field in an insulated box equipped with a Phase Change Material (PCM) under various PCM position, aspect ratio of the box, ambient temperature, initial load temperature (test product, Tylose) and space beneath the load. Table 1 summarizes 10 experimental conditions presented in this article and their corresponding figures.

**Table 1:** Experimental conditions.

Condition	Pictogram	PCM position	Aspect ratio (height/width)	Ambient temperature (°C)	Initial load temperature (°C)	Spacing beneath load (mm)	Corresponding figures
1		Side wall	1.0	20	4	20	1
2		Top	1.0	20	4	20	2
3		Side wall	1.7	20	4	20	3
4		Side wall	1.0	30	4	20	4 and 5
5		Top	1.0	10	4	20	6
6		Top	1.0	30	4	20	7
7		Side wall	1.0	20	10	20	8 and 9
8		Top	1.0	20	10	20	10
9		Side wall	1.0	20	4	0	11
10		Top	1.0	20	4	0	12

The air velocity component, its magnitude and uncertainty of measurement under these conditions are shown in Dataset 1: Air velocity in an insulated box (https://data.mendeley.com/datasets/ggmkzk634h/1)

The average temperature in the insulated box under these conditions are shown in Dataset 2: Average temperature in an insulated box (https://data.mendeley.com/datasets/sz5dgkz7k8/1)

The temperature contour field was drawn via MATLAB by interpolating the measured temperature at 30 positions during stable conditions. The codes of this drawing are shown in Tanathepl/Temperature-contour

(https://github.com/Tanathepl/Temperature-contour.git)

**Fig. 1:**
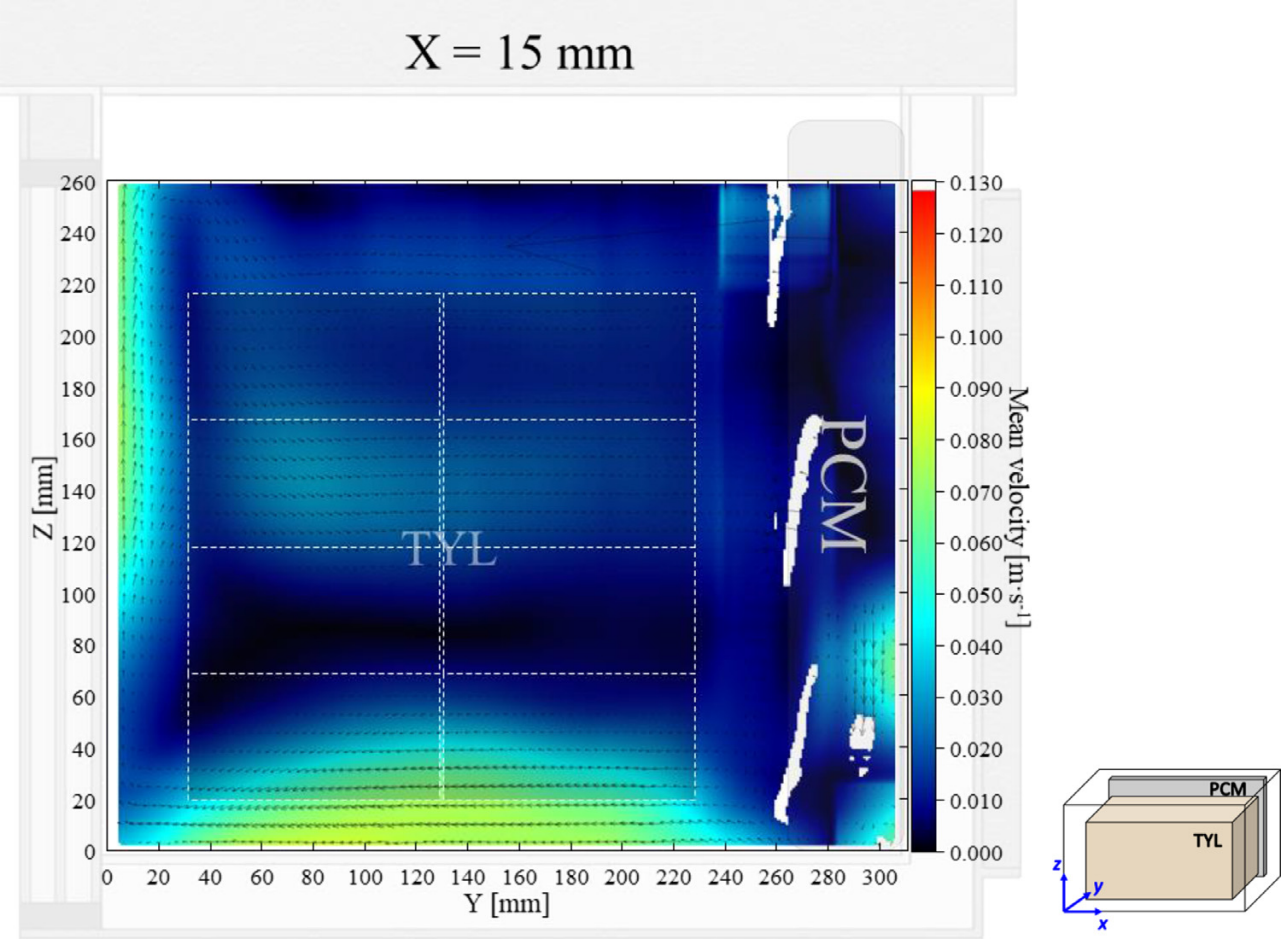
Air velocity field at *X* = 15 mm in a loaded horizontal box with PCM on a sidewall under 20 °C ambient, product initial temperature = 4 °C with 20 mm gap below. Note: unmeasured air velocity in white areas on the right can be explained by the refraction from PCM surface behind the laser sheet.

**Fig. 2:**
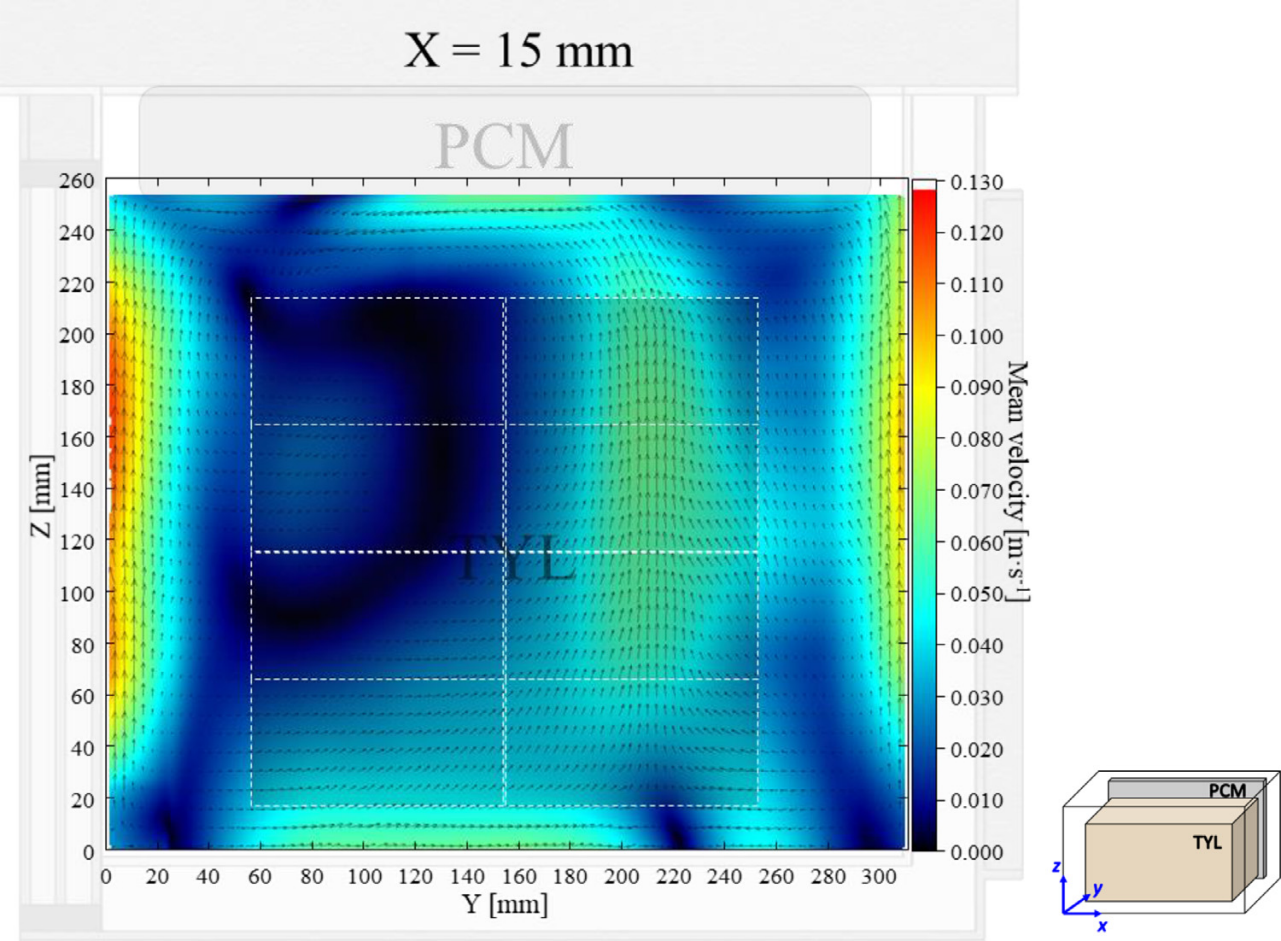
Air velocity field at *X* = 15 mm in a loaded horizontal box with PCM at the top under 20 °C ambient, product initial temperature = 4 °C with 20 mm gap below.

The air velocity field at *X* = 15 mm in a loaded horizontal box (aspect ratio ∼ 1) with PCM on a sidewall under 20 °C ambient, product initial temperature = 4 °C with 20 mm gap below (condition 1) are shown in Fig. 1. Air velocity and temperature fields at *X* = 250 mm are not shown here, they can be found in Leungtongkum et al. [1] (Figs. 2a and 2a’).

The air velocity field at *X* = 15 mm in a loaded horizontal box with PCM at the top under 20 °C ambient, product initial temperature = 4 °C with 20 mm gap below (condition 2) are shown in Fig. 2. Air velocity and temperature fields at *X* = 250 mm are not shown here, they can be found in Leungtongkum et al. [1] (Fig. 2b and b’).

**Fig. 3:**
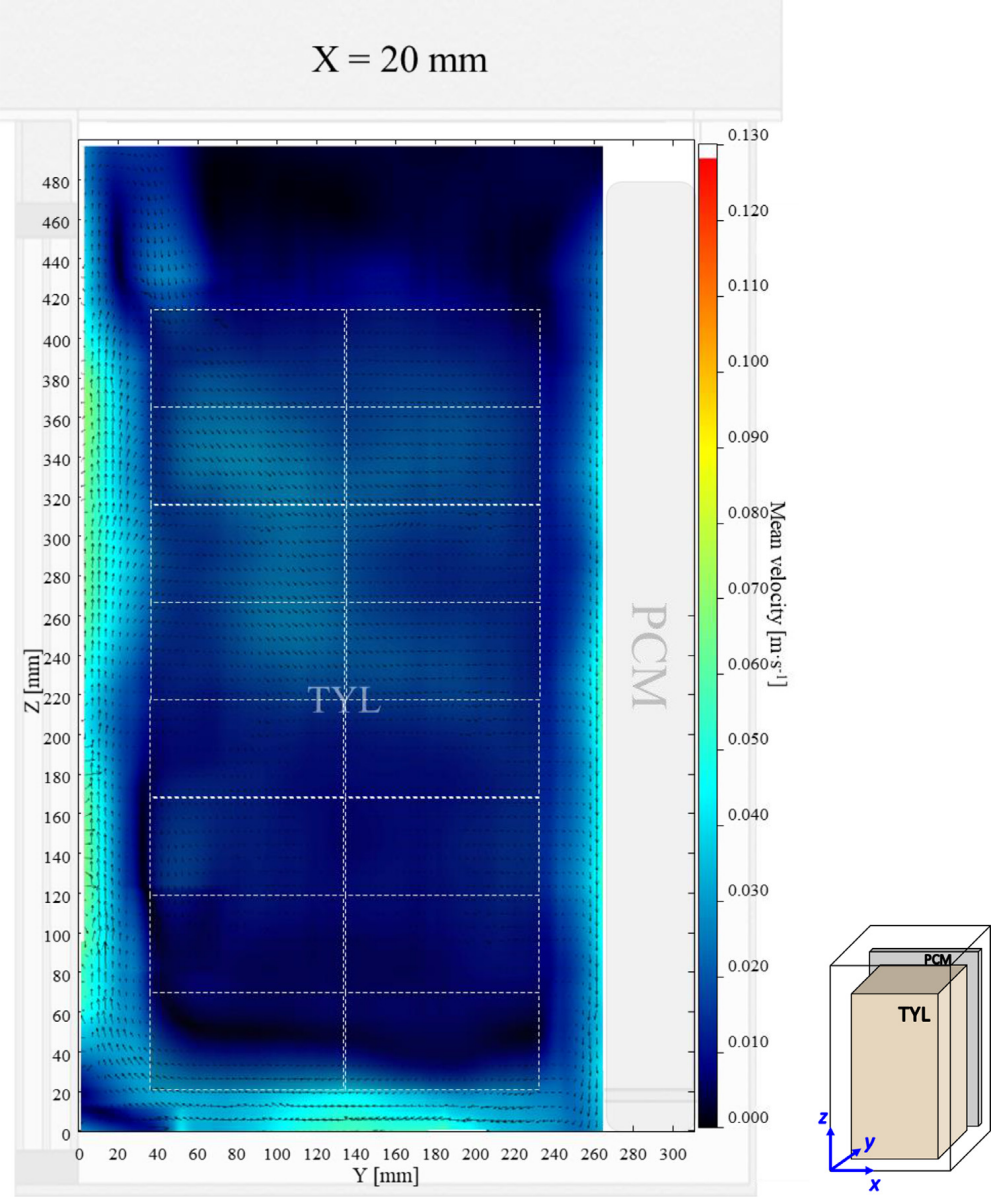
Air velocity field at *X* = 20 mm in a loaded vertical box with PCM on a sidewall under 20 °C ambient, product initial temperature = 4 °C with 20 mm gap below.

The air velocity field at *X* = 20 mm in a loaded vertical box (aspect ratio = 1.7) with PCM on a sidewall under 20 °C ambient, product initial temperature = 4 °C with 20 mm gap below (condition 3) are shown in Fig. 3. Air velocity and temperature fields at *X* = 250 mm are not shown here, they can be found in Leungtongkum et al. [1] (Fig. 2c and c’).

**Fig. 4:**
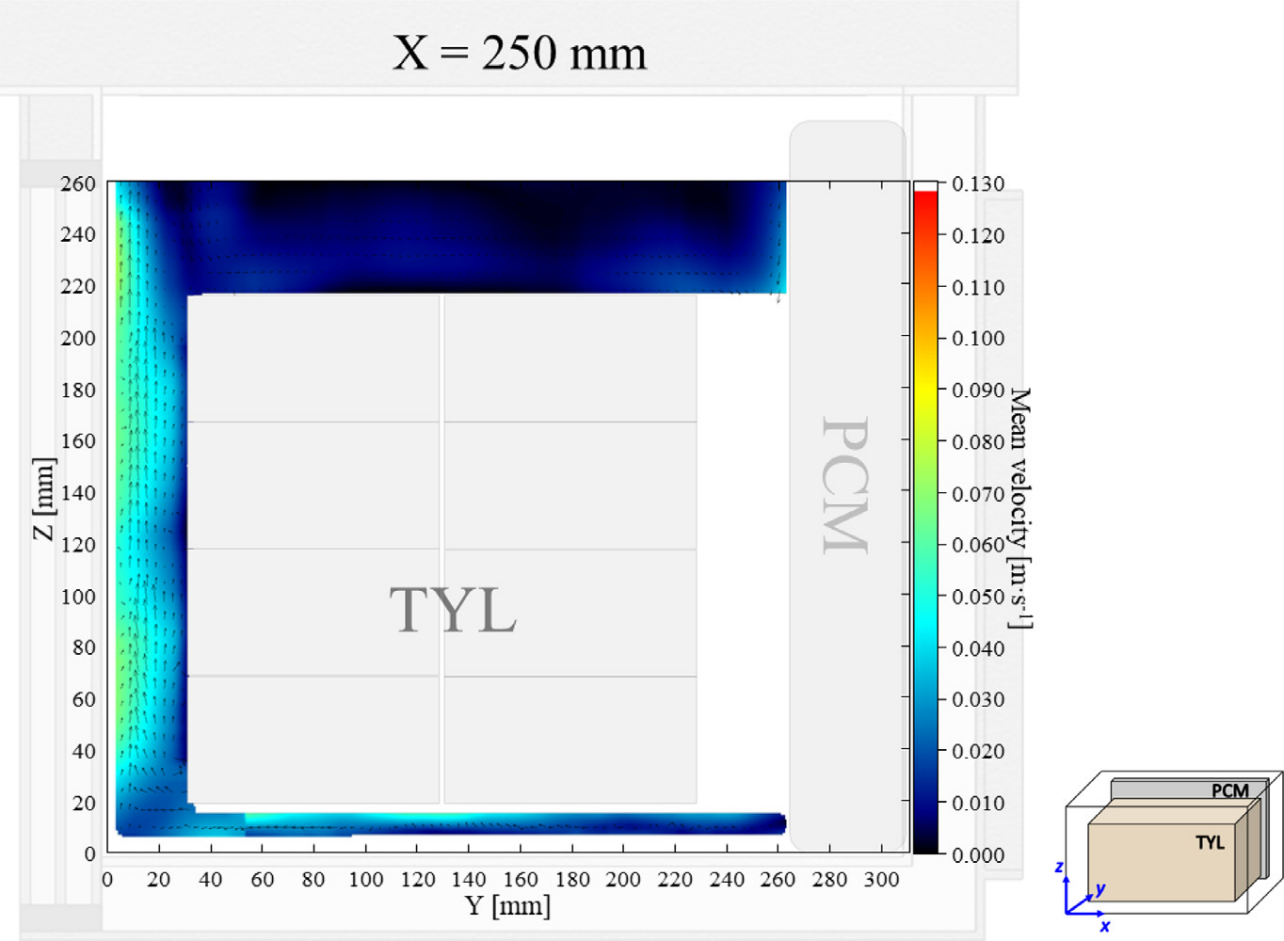
Air velocity field at *X* = 250 mm in a loaded horizontal box with PCM on a sidewall under 30 °C ambient, product initial temperature = 4 °C with 20 mm gap below. White area on the right represents the unmeasurable zone because of the inaccessibility of laser sheet.

**Fig. 5:**
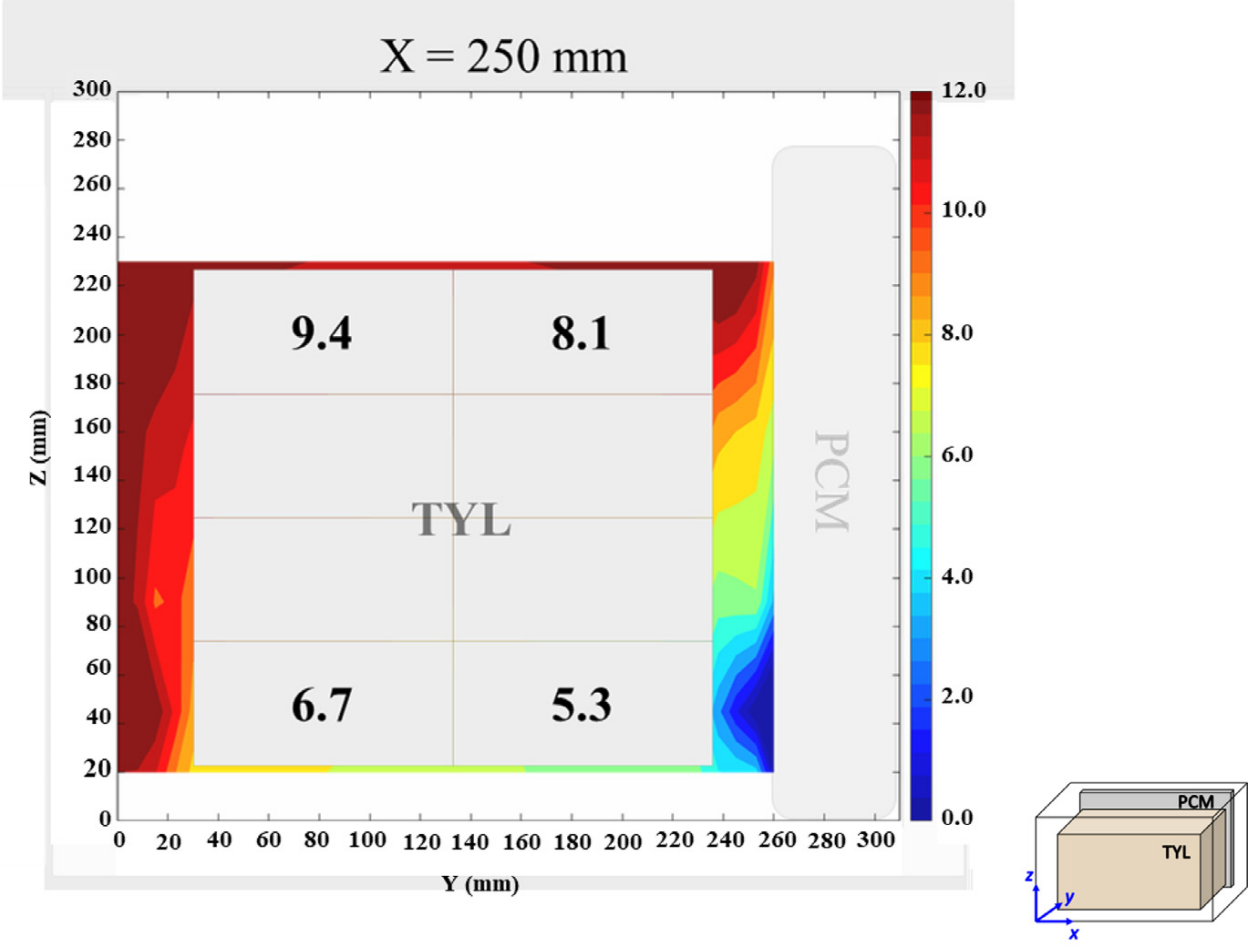
Air temperature field and core temperature of test product ( °C) at *X* = 250 mm in a loaded horizontal box with PCM on a sidewall under 30 °C ambient, product initial temperature = 4 °C with 20 mm gap below.

The air velocity and temperature fields at *X* = 250 mm in a loaded horizontal box with PCM on a sidewall under 30 °C ambient, product initial temperature = 4 °C with 20 mm gap below (condition 4) are shown in Fig. 4, Fig. 5.

**Fig. 6:**
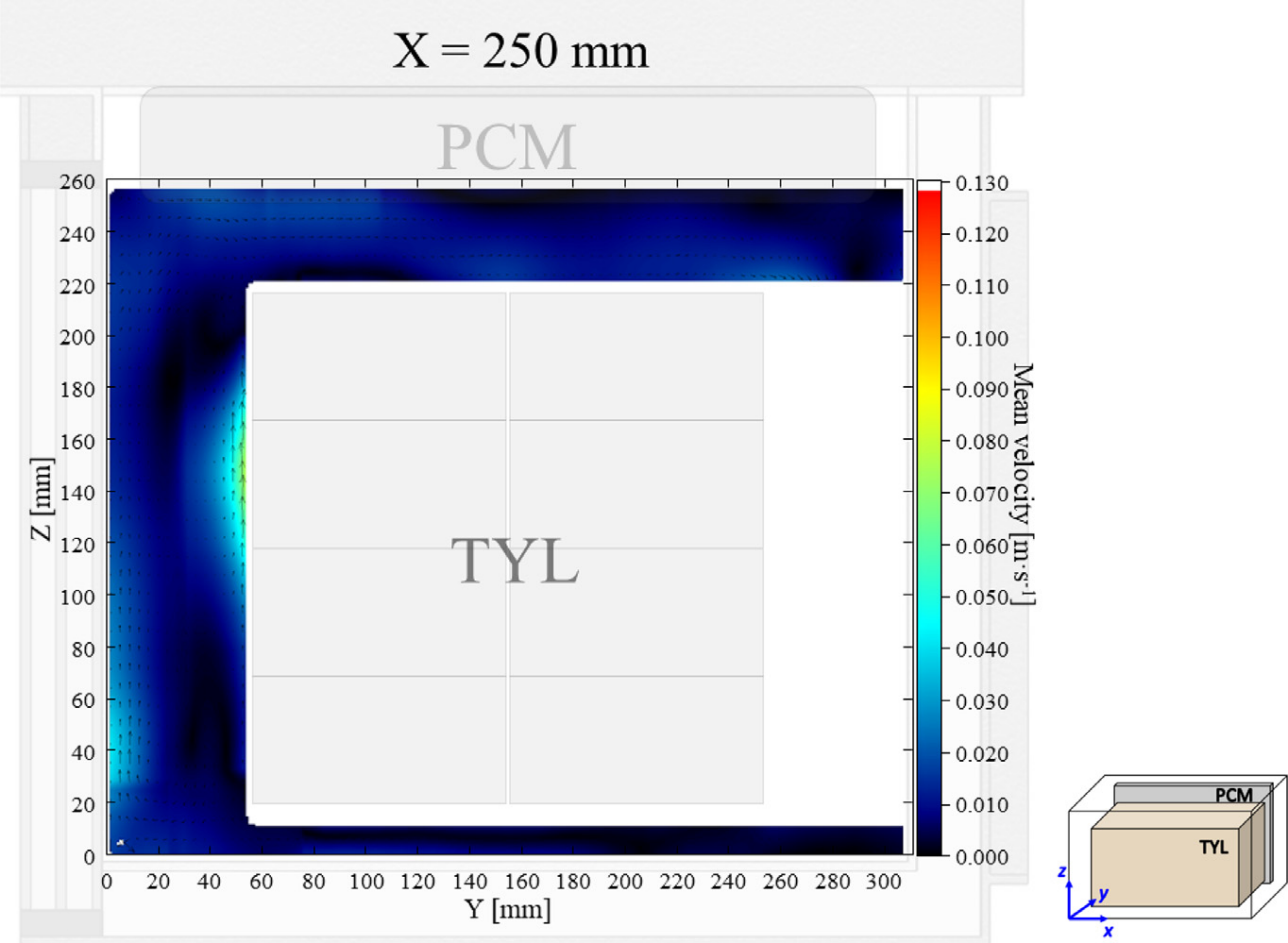
Air velocity field on the middle plane in a loaded horizontal box with PCM at the top under 10 °C ambient, product initial temperature = 4 °C with 20 mm gap below. White area on the right represents the unmeasurable zone because of the inaccessibility of laser sheet.

The air velocity field at *X* = 250 mm in a loaded horizontal box with PCM at the top under 10 °C ambient, product initial temperature = 4 °C with 20 mm gap below (condition 5) are shown in Fig. 6. Temperature field at *X* = 250 mm are shown in Fig. 3a of Leungtongkum et al. [1].

**Fig. 7:**
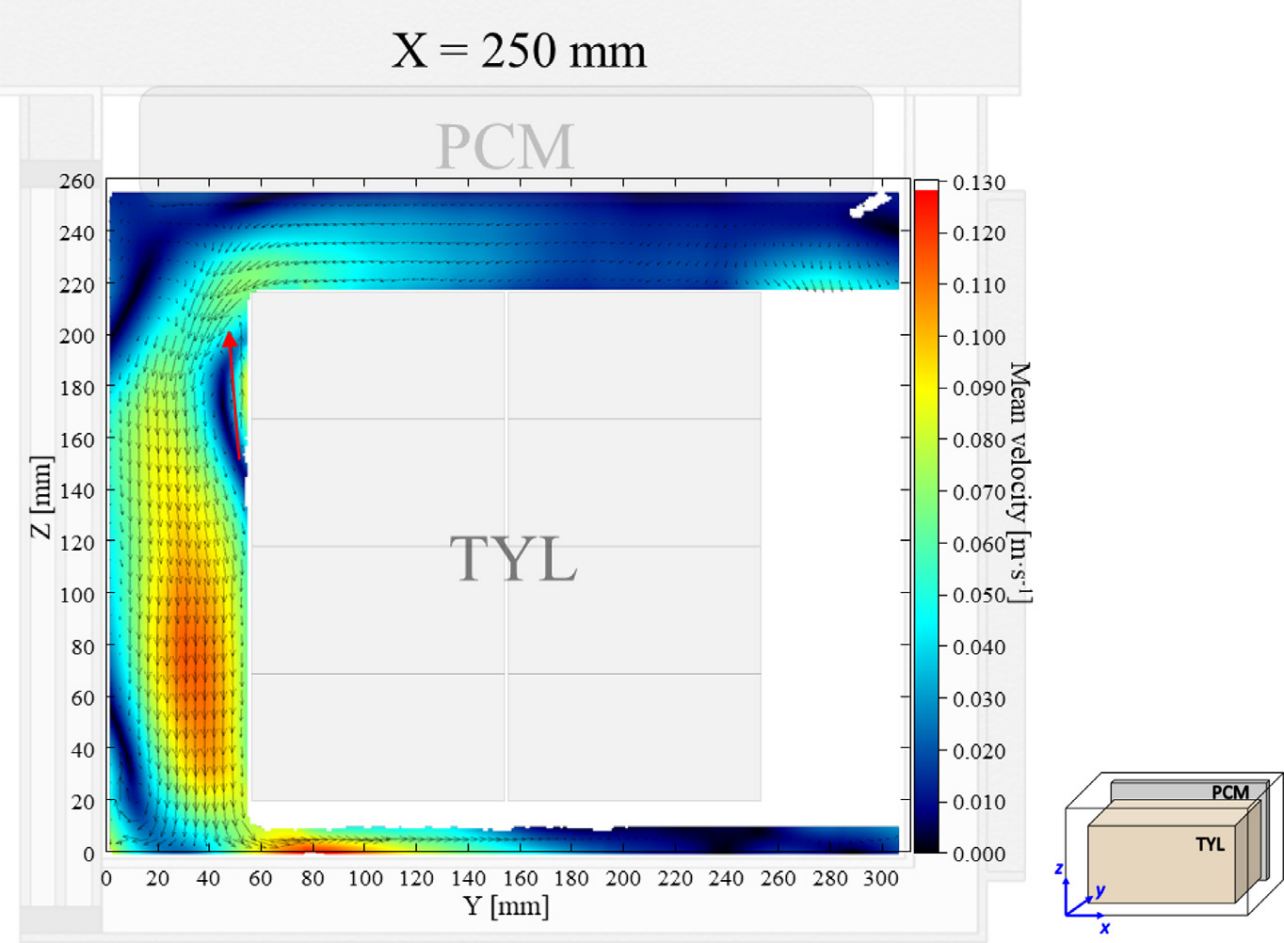
Air velocity field on the middle plane in a loaded horizontal box with PCM at the top under 30 °C ambient, product initial temperature = 4 °C with 20 mm gap below. White area on the right represents the unmeasurable zone because of the inaccessibility of laser sheet.

The air velocity field at *X* = 250 mm in a loaded horizontal box with PCM at the top under 30 °C ambient, product initial temperature = 4 °C with 20 mm gap below (condition 6) are shown in Fig. 7. Temperature field at *X* = 250 mm are shown in Fig. 3c of Leungtongkum et al. [1].

**Fig. 8:**
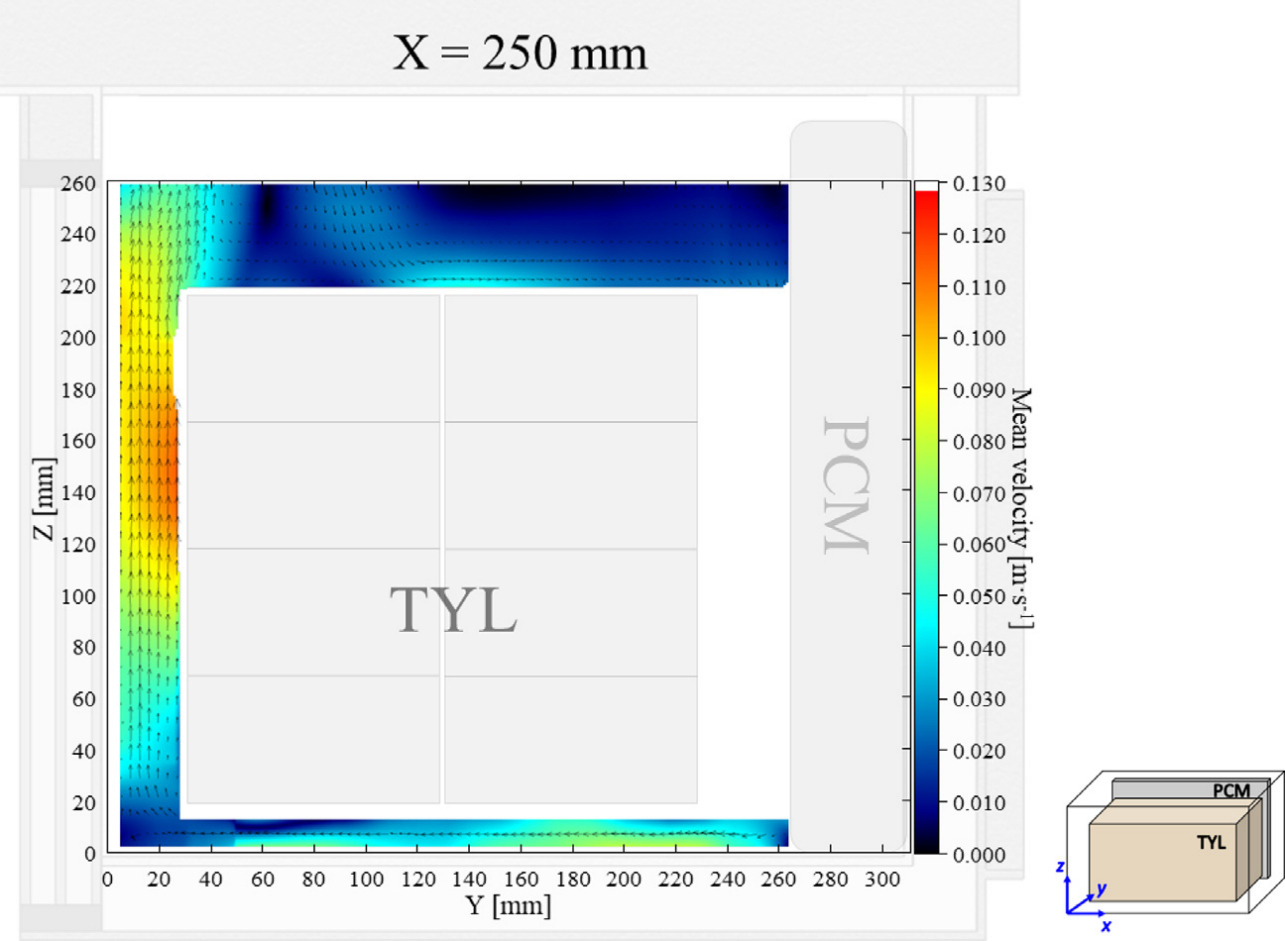
Air velocity field on the middle plane in a loaded horizontal box with PCM on a sidewall under 20 °C ambient, product initial temperature = 10 °C with 20 mm gap below. White area on the right represents the unmeasurable zone because of the inaccessibility of laser sheet.

**Fig. 9:**
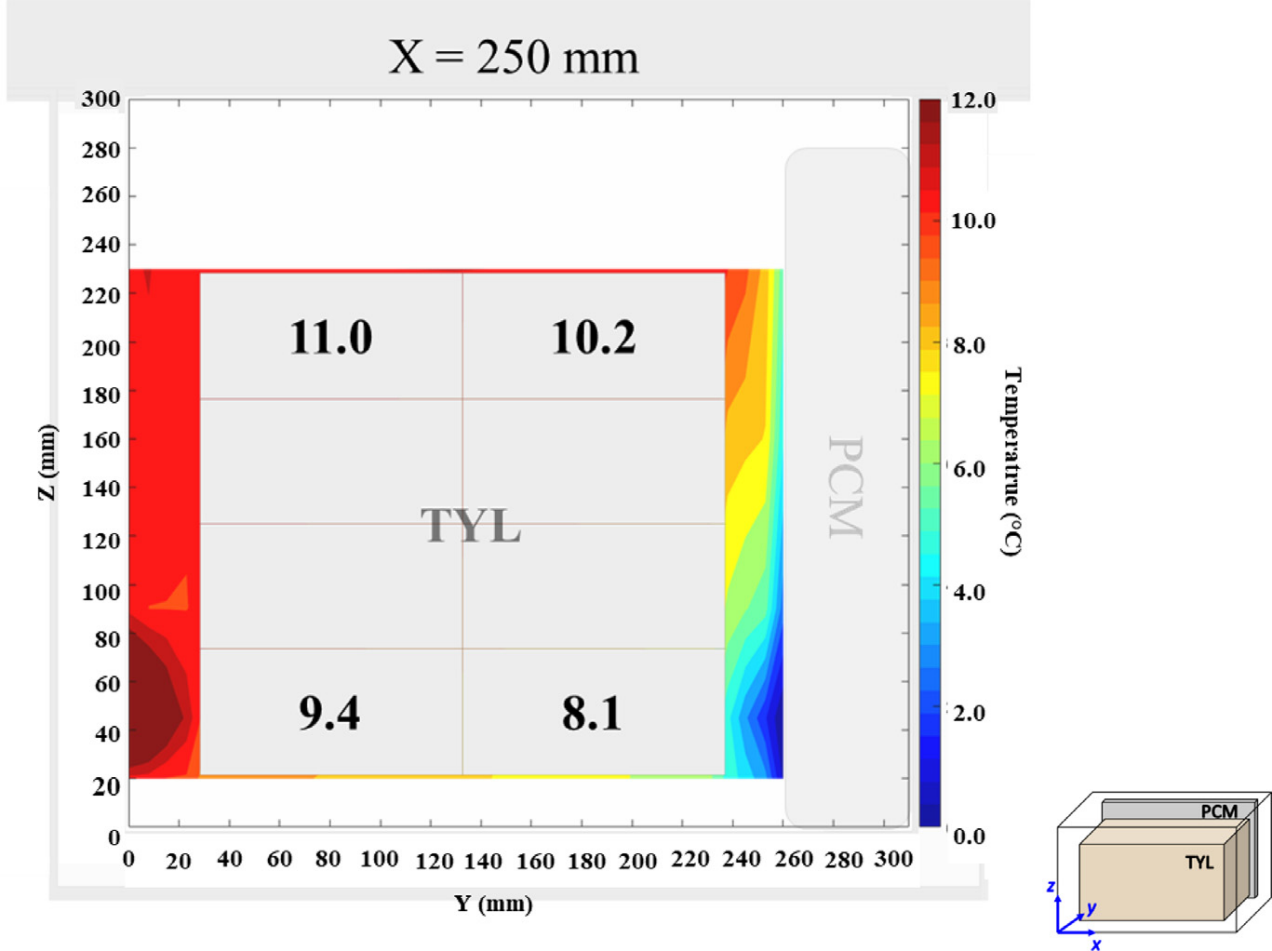
Air temperature field and core temperature of test product (°C) on the middle plane in a loaded horizontal box with PCM on a sidewall under 20 °C ambient, product initial temperature = 10 °C with 20 mm gap below.

The air velocity field and temperature field at *X* = 250 mm in a loaded horizontal box with PCM on a sidewall under 20 °C ambient, product initial temperature = 10 °C with 20 mm gap below (condition 7) are shown in Fig. 8, Fig. 9.

**Fig. 10:**
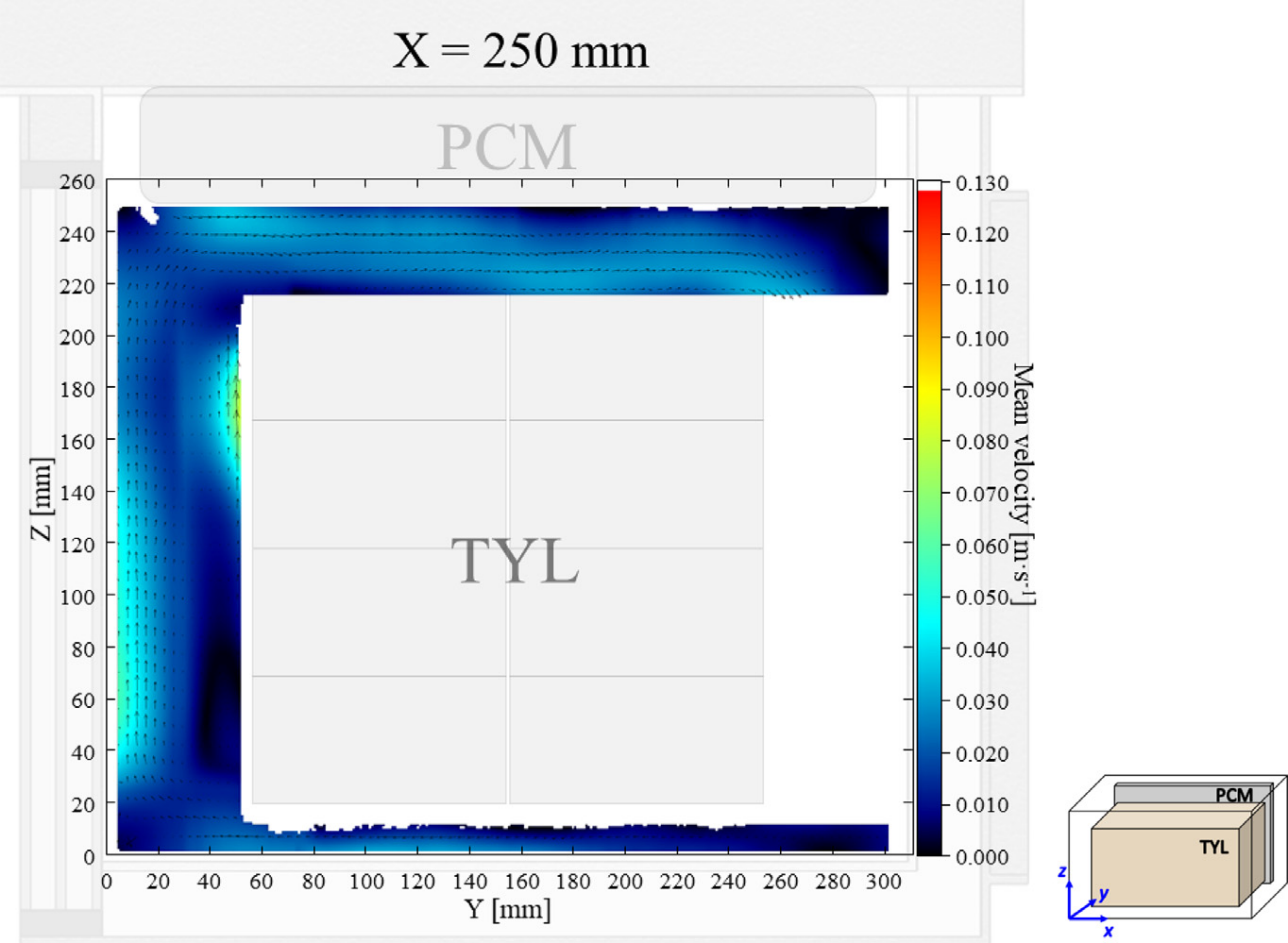
Air velocity field on the middle plane in a loaded horizontal box with PCM at the top under 20 °C ambient, product initial temperature = 10 °C with 20 mm gap below. White area on the right represents the unmeasurable zone because of the inaccessibility of laser sheet.

The air velocity field at *X* = 250 mm in a loaded horizontal box with PCM at the top under 20 °C ambient, product initial temperature = 10 °C with 20 mm gap below (condition 8) are shown in Fig. 10. Temperature field at *X* = 250 mm are not shown here, it can be found in Leungtongkum et al. [1] (Fig. 3d).

**Fig. 11:**
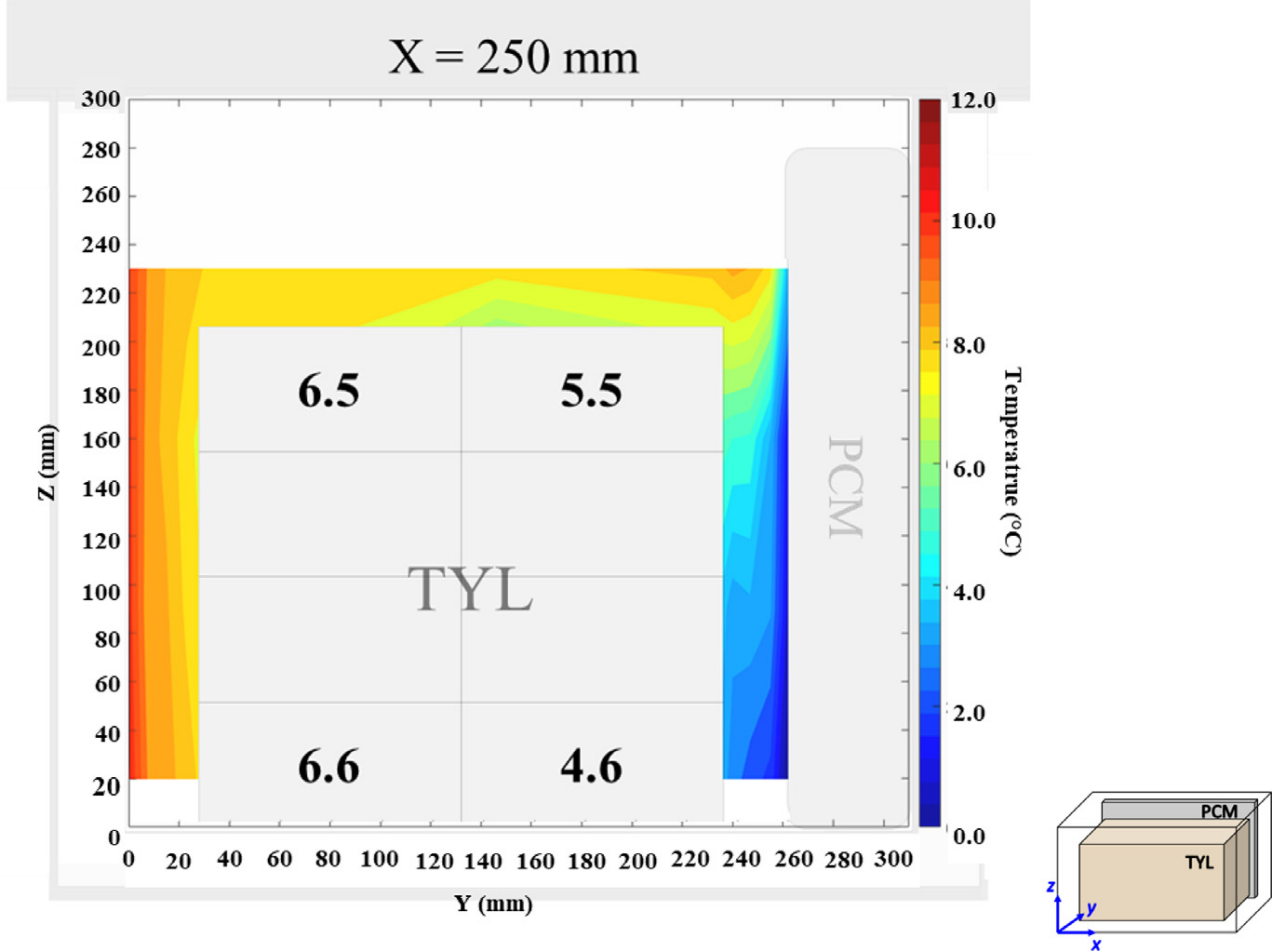
Air temperature field and core temperature of test product ( °C) on the middle plane in a loaded horizontal box with PCM on a sidewall under 20 °C ambient, product initial temperature = 4 °C without gap below.

The temperature field at *X* = 250 mm in a loaded horizontal box with PCM on a sidewall under 20 °C ambient, product initial temperature = 4 °C without gap below (condition 9) are shown in Fig. 11. Air velocity field at *X* = 250 mm are shown not shown here, it can be found in Leungtongkum et al. [1] (Fig. 4d).

**Fig. 12:**
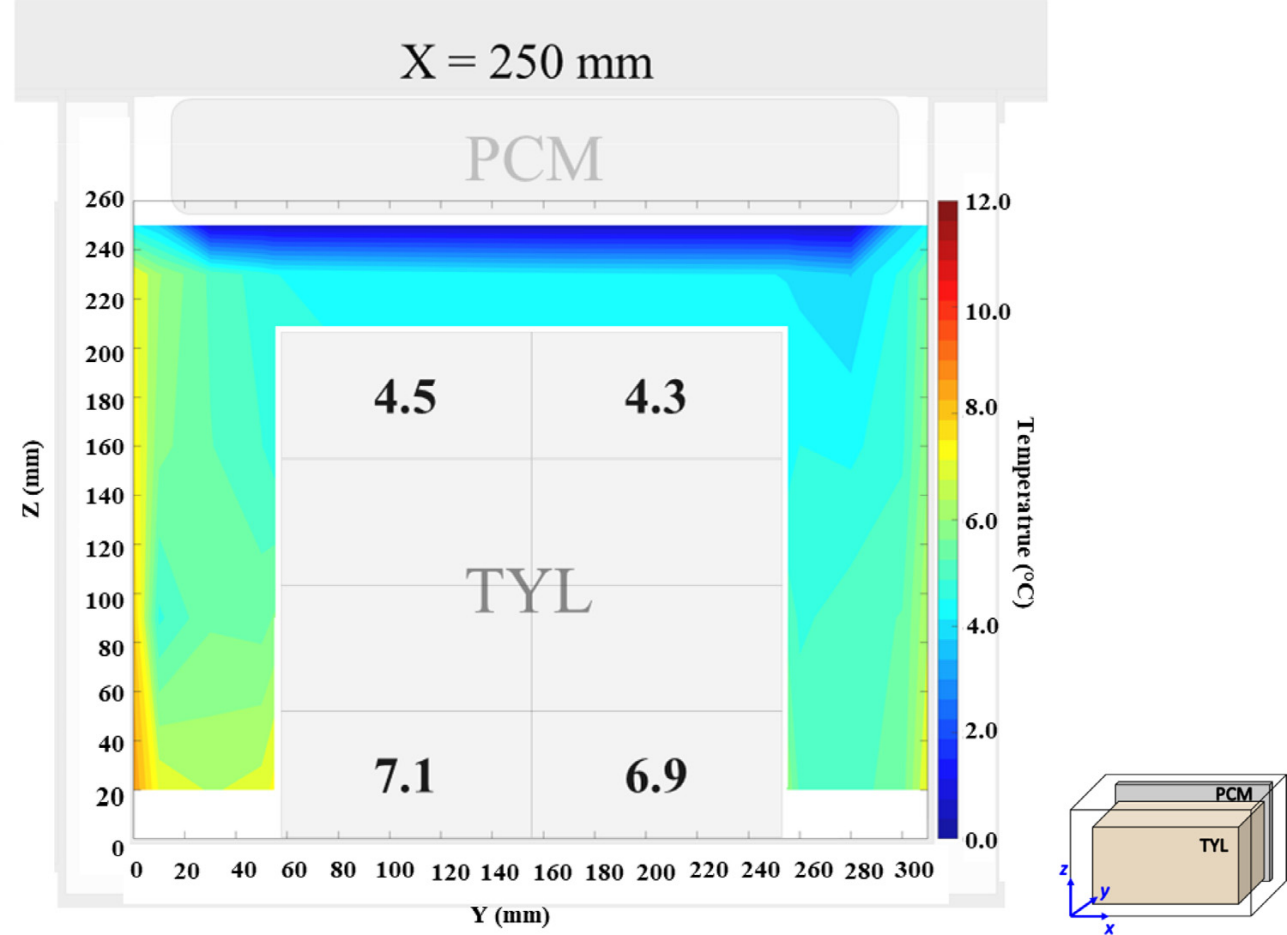
Air temperature field and core temperature of test product (°C) on the middle plane in a loaded horizontal box with PCM at the top under 20 °C ambient, product initial temperature = 4 °C without gap below.

The temperature field at *X* = 250 mm in a loaded horizontal box with PCM at the top under 20 °C ambient, product initial temperature = 4 °C without gap below (condition 10) are shown in Fig. 12. Air velocity field at *X* = 250 mm are not shown here, it can be found in Leungtongkum et al. [1] (Fig. 4b).

## 3 Experimental Design, Materials and Methods

The material and methods described in detailed in Leungtongkum et al. [2] are shown succinctly below.

### 3.1 Material

The details of material and experimental setup description can be found in Leungtongkum et al. [2].

### 3.2 Thermal study

The box was loaded with 16 test product packs initially at 4 °C or 10 °C and a PCM slab initially at −2 °C. The temperature measurement of product, air, internal wall and PCM was carried out with 34 thermocouples (T-type thermocouples) at the middle plane (*x* = 250 mm). The diagram showing thermocouples positions is in Fig. 1 of Leungtongkum et al. [1]. The measurement started after the box closing until complete PCM melted without the box opening during the experiment during which the temperatures were recorded continuously (every 30 s) using Agilent 34972A data acquisition unit (Agilent Technologies, CA, USA). The results of the stabilization period ranging from 400 min. to 600 min. were analyzed and compared. This period was chosen based on temperature variations were less than 0.3 °C for all conditions. The temperature contour map was drawn by MATLAB with interpolation. More details of experimental setup for temperature measurement can be found in Leungtongkum et al. [2].

**Fig. 13:**
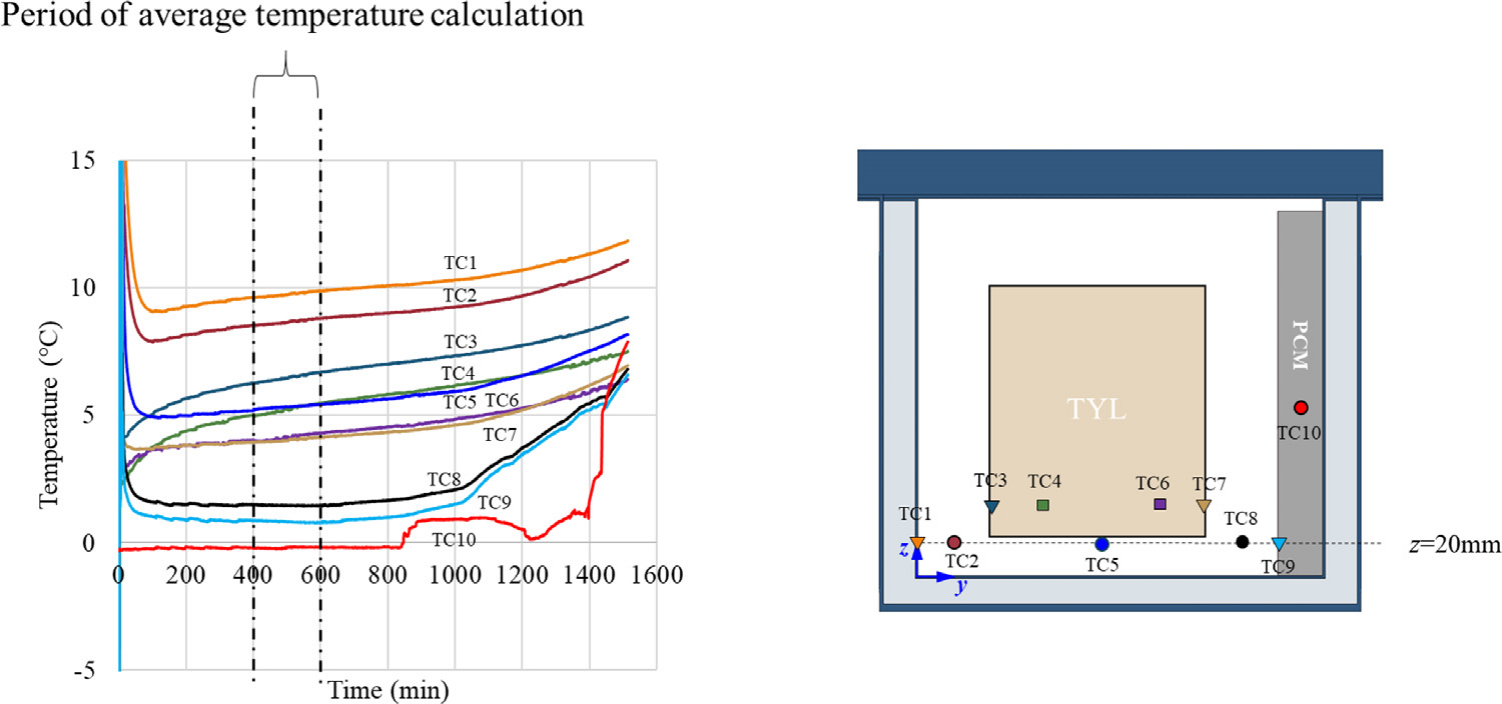
Temperature evolution at the bottom of a loaded horizontal box with PCM at sidewall under 20 °C ambient, product initial temperature = 4 °C with 20 mm gap below.

Fig. 13 shows an example of temperature evolution at the bottom of a loaded horizontal box with PCM on a sidewall under 20 °C ambient, product initial temperature = 4 °C (condition 1). The average temperatures calculated from 400 min to 600 min were shown in Figs. 5, 9, 11 and 12 to present the temperature field in stable condition.

### 3.3 Airflow study

#### 3.3.1 Instrumentation

The PIV device is constituted of three parts: a double-pulsed Nd:YLF laser (527 nm wavelength, 10 mJ pulse energy), a high-speed 12-bit CMOS video camera (Photron, FASTCAM SA3; 1024 × 1024 pixels in resolution) fitted with a lens (Sigma; 105 mm, f/1:2.8) and a programmable timing unit (PTU-X) to guarantee synchronization of the laser and the camera. Visualization of the airflow pattern is possible by the scattering of smoke particle during laser pulses. Oil-based particles (mean diameter 0.3 μm) were generated using a smoke machine (Antari, F-80Z). More detail on PIV system can be found in Leungtongkum et al. [2].

More details of image acquisition, image post-processing and experimental protocol can be found in Leungtongkum et al. [2].

## CRediT authorship contribution statement

**Tanathep Leungtongkum:** Conceptualization, Methodology, Investigation, Validation, Formal analysis, Software, Writing – original draft, Visualization. **Denis Flick:** Methodology, Validation, Formal analysis, Writing – review & editing, Supervision. **Nattawut Chaomuang:** Conceptualization, Methodology, Investigation, Validation, Formal analysis, Software, Writing – original draft, Visualization, Funding acquisition. **Alain Denis:** Investigation, Software. **Onrawee Laguerre:** Validation, Formal analysis, Writing – review & editing, Supervision, Project administration, Funding acquisition.

## Data Availability

Air velocity in an insulated box (Original data) (Mendeley Data) Temperature-contour (Original data) (GitHub) Average temperature in an insulated box (Original data) (Mendeley Data)
